# Management of Intracranial Meningiomas Using Keyhole Techniques

**DOI:** 10.7759/cureus.588

**Published:** 2016-04-27

**Authors:** Joshua D Burks, Andrew K Conner, Phillip A Bonney, Jacob B Archer, Blake Christensen, Jacqueline Smith, Sam Safavi-Abbasi, Michael Sughrue

**Affiliations:** 1 Department of Neurosurgery, University of Oklahoma Health Sciences Center; 2 Department of Neurosurgery, Indiana University School of Medicine; 3 Department of Anesthesiology, University of Oklahoma Health Sciences Center

**Keywords:** meningioma, keyhole, retrosigmoid, pterional, supraorbital, eyebrow, craniotomy, tumor resection

## Abstract

Background: Keyhole craniotomies are increasingly being used for lesions of the skull base. Here we review our recent experience with these approaches for resection of intracranial meningiomas.

Methods: Clinical and operative data were gathered on all patients treated with keyhole approaches by the senior author from January 2012 to June 2013. Thirty-one meningiomas were resected in 27 patients, including 9 supratentorial, 5 anterior fossa, 7 middle fossa, 6 posterior fossa, and 4 complex skull base tumors. Twenty-nine tumors were WHO Grade I, and 2 were Grade II.

Results: The mean operative time was 8 hours, 22 minutes (range, 2:55-16:14) for skull-base tumors, and 4 hours, 27 minutes (range, 1:45-7:13) for supratentorial tumors. Simpson Resection grades were as follows: Grade I = 8, II = 8, III = 1, IV = 15, V = 0. The median postoperative hospital stay was 4 days (range, 1-20 days). In the 9 patients presenting with some degree of visual loss, 7 saw improvement or complete resolution. In the 6 patients presenting with cranial nerve palsies, 4 experienced improvement or resolution of the deficit postoperatively. Four patients experienced new neurologic deficits, all of which were improved or resolved at the time of the last follow-up. Technical aspects and surgical nuances of these approaches for management of intracranial meningiomas are discussed.

Conclusions: With careful preoperative evaluation, keyhole approaches can be utilized singly or in combination to manage meningiomas in a wide variety of locations with satisfactory results.

## Introduction

The keyhole concept has been increasingly utilized in the neurosurgical community as a safe and effective means of resecting intracranial lesions while minimalizing exposure of other structures [1-3]. While individual approaches have been described, exact paradigms for approach selection for specific tumors are ill-defined. Further, there is a paucity of literature on the use of keyhole techniques in the resection of intracranial meningiomas. Here we describe the resection of 31 intracranial meningiomas in 27 patients, all performed through various tailored keyhole craniotomies.

## Materials and methods

**Figure 1:**
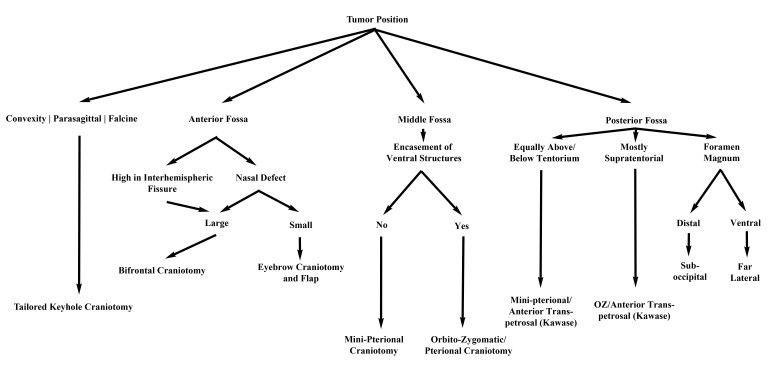
Our treatment paradigm for new meningiomas

This retrospective study was approved by the University of Oklahoma Health Sciences Center Institutional Review Board (IRB #3199, including waiver of consent). We queried departmental records for all patients with meningiomas treated by the senior author (MES) at our institution from January 2012 to June 2013. We reviewed clinical records and imaging of 44 patients; the data collected included demographics, location of lesion(s), approach used, operative time, Simpson grade of resection, WHO grade of lesion(s), postoperative length of hospital stay, perioperative complications, adjuvant therapy, and postoperative outcome. Patients were grouped according to their surgical approach - supraorbital, mini-pterional, mini-retrosigmoid, tailored craniotomy (for supratentorial tumors), or a combination of approaches. Figure 1 presents our method for selecting an appropriate approach. All patients had at least 1 postoperative follow-up visit after 1-2 weeks, and all but 1 underwent postoperative magnetic resonance imaging (MRI) with gadolinium administration performed at that time. This patient had a previous ferrous metal implant precluding MRI and was evaluated with CT instead.

### Supraorbital 'eyebrow' approach

**Figure 2:**
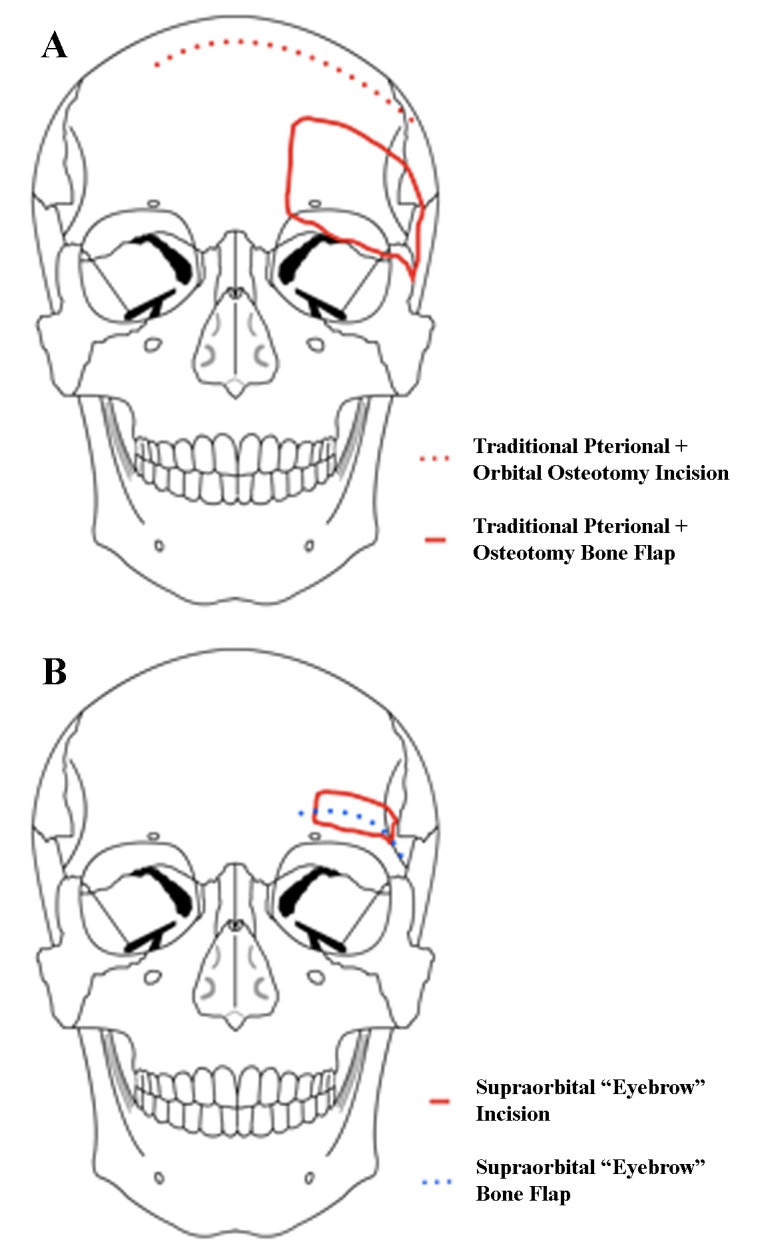
Standard pterional and orbital osteotomy approach (A) vs. supra-orbital approach (B) In a supraorbital approach, an incision is made within the eyebrow and lateral to the supraorbital nerve, and the frontalis muscle is divided parallel to the orbital rim and reflected downward. The keyhole is made just posterior to the temporal line, and the orbital rim is drilled flush with the orbital roof. Modified from: Rosario Van Tulpe, https://commons.wikimedia.org/wiki/File:SkullSchaedel3.png

The senior author uses the supraorbital 'eyebrow' craniotomy as a workhorse for the majority of anterior fossa and some middle fossa meningiomas. The patient is positioned supine on the operating table; the head is typically elevated 15°, extended 10-20°, and rotated to the contralateral side between 10-80° depending on the location of the tumor (anterior tumors like olfactory groove meningiomas require more rotation). The head is also flexed laterally by about 10° to provide a more ergonomic work position for the surgeon. The incision is made within the eyebrow, lateral to the supraorbital nerve. The subcutaneous tissue is dissected down and the frontalis muscle divided parallel to the orbital rim. The frontalis muscle is carefully elevated off pericranium, which is reflected downward. A burr hole is placed in the keyhole area, posterior to the temporal line, and a small craniotomy (15-20 mm in width, 10-15 mm in height) is created. This may be made superomedially to the burr hole or more laterally toward the pterion depending on the location of the lesion and the desired angle of attack. The inner edge of the craniotomy defect above the orbital rim is drilled flush with the orbital roof; in the latter variation, the lateral aspect of the lesser sphenoid wing is also drilled down. Depending on the location of the lesion, the dura can be opened in a curved fashion following the superior aspect of the craniotomy. When the dural opening is flush with the orbital roof, significant frontal lobe retraction is typically unnecessary. A diagram is provided in Figure 2 to contrast the difference between the opening for this approach and the standard pterional and orbital osteotomy approach.

The operating microscope is introduced and the anterior fossa floor followed to the cisterns, draining CSF early and dissecting the arachnoid to find the site of the tumor. The optic nerves and anterior circulation vessels are identified and the dural base is coagulated before dissecting the tumor away from these structures. Once the lesion is satisfactorily resected, we perform a watertight closure with or without a dural graft. Although it occurs rarely, if the frontal sinus is inadvertently entered, it is repaired meticulously. The bone flap is replaced with titanium plating system, the pericranium and frontalis muscle closed in two layers, followed by closure of the skin in a cosmetic fashion using a subcuticular nylon stitch.

### Mini-pterional approach

**Figure 3:**
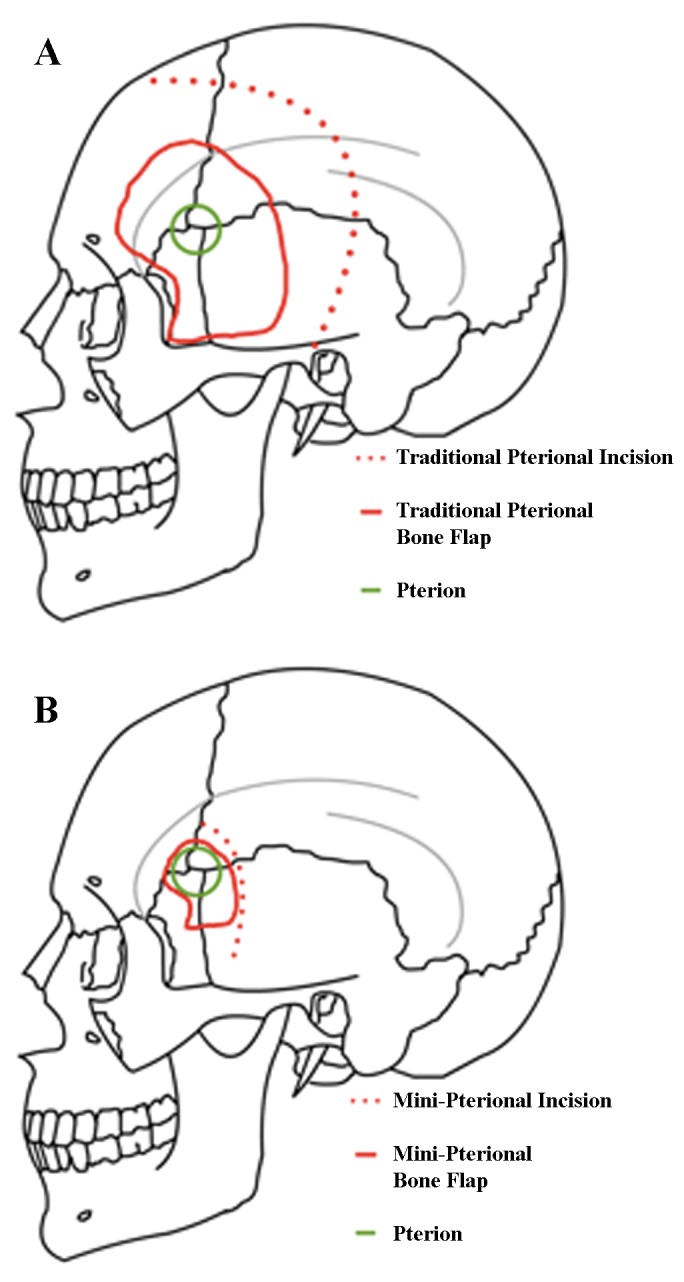
Standard pterional approach (A) vs. mini-pterionaly approach (B) The incision of a mini-pterional approach is made approximately 1 cm superior to the zygomatic arch and 1 cm anterior to the external auditory meatus, curving around the normal hairline and ending at the midline. The scalp flap and superficial fat pad are elevated together and reflected anteriorly, with care taken to preserve the superficial temporal artery and frontal branches of the facial nerve. The temporalis muscle is reflected inferiorly with subperiosteal dissection to preserve the deep temporal nerves and vasculature to reveal the pterion. In contrast to the traditional craniotomy where the temporalis is reflected anteriorly, it is cleared away from the sphenoid wing. The posterior portions of the temporalis muscle are left intact. Modified from: Rosario Van Tulpe, https://commons.wikimedia.org/wiki/File:SkullSchaedelSeitlich1.png

The patient is positioned supine in the typical pterional fashion. The incision is made approximately 1 cm superior to the zygomatic arch and 1 cm anterior to the external auditory meatus, curving around the normal hairline and ending at the midline. The scalp flap and superficial fat pad are elevated together and reflected anteriorly; either interfascial or subfascial dissection may be performed at this point to protect the frontal branches of the facial nerve. The temporalis muscle is cut in the plane of its fibers directly at the pterion and reflected inferiorly with subperiosteal dissection to preserve the deep temporal nerves and vasculature, revealing the pterion. Differences from a traditional pterional opening are illustrated in Figure 3. Miniaturization of a traditional craniotomy requires that the temporalis muscle be cleared out of the way of the sphenoid wing, instead of reflected anteriorly. The posterior portions of the temporalis muscle are left intact. A burr hole is placed above the frontozygomatic suture inferior to the linea temporalis. A craniotomy is then created to include the lateral aspect of the sphenoid, part of the frontal bone inferior to the superior temporal line, and a minimal portion of the temporal bone. The sphenoid ridge is drilled down to the depth of the superior orbital fissure. We generally perform extensive extradural work as needed, including clinoidectomy, extradural peeling of the lateral cavernous sinus, and cauterization of the middle meningeal artery. All of these maneuvers can be done easily through a well planned mini-pterional craniotomy.

The dura is opened in a curvilinear fashion with the base of the flap directed toward the skull base. From here the inferolateral frontal lobe, Sylvian fissure, and superior temporal gyrus are evident. The Sylvian fissure is microsurgically dissected in the usual fashion to expose the tumor, optic nerves and chiasm, and the anterior circulation vessels. The dural attachments are identified and coagulated, and the tumor is dissected free of vital neurovascular structures. Care is taken to dissect the tumor from surrounding structures within the subarachnoid plane. Additionally, bony hyperostosis may be removed with high-speed drilling, if not drilled extradurally. Once the meningioma has been satisfactorily resected, the dura is closed with or without a dural patch.

### Mini-retrosigmoid approach

**Figure 4:**
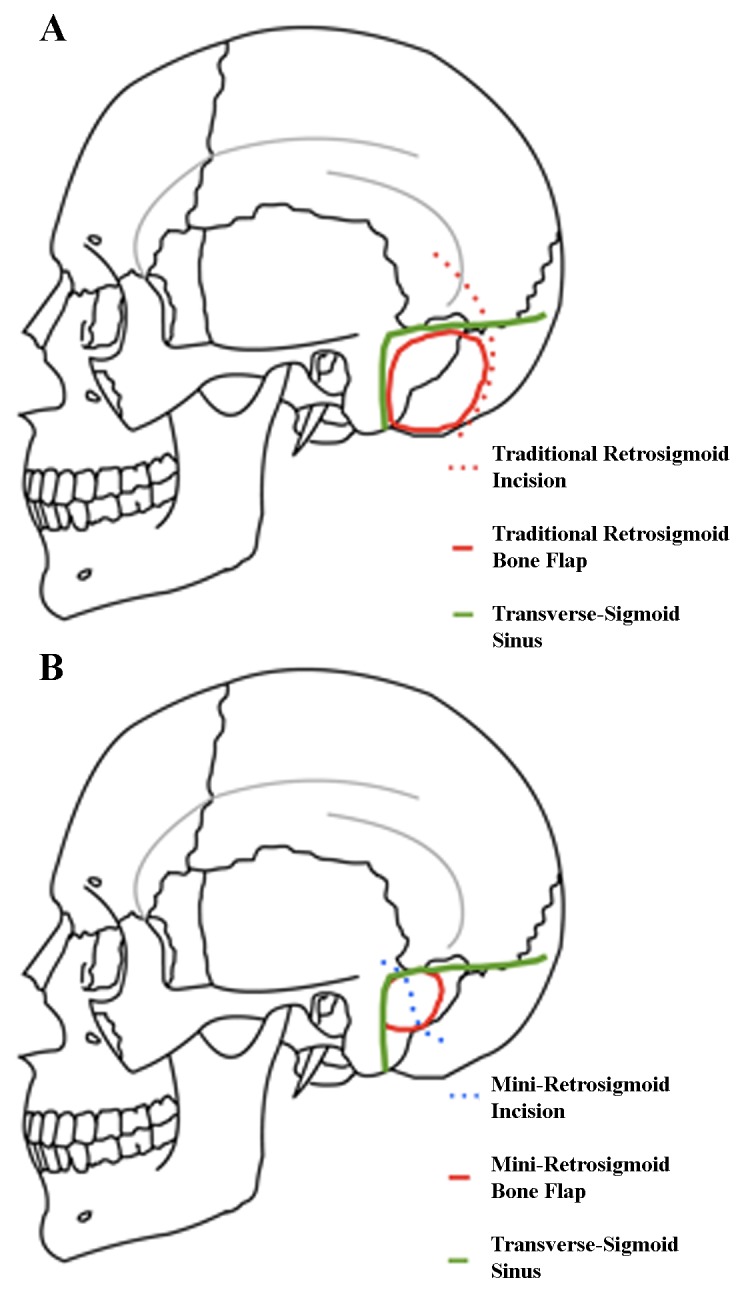
Standard retrosigmoid approach (A) vs. mini-retrosigmoid approach (B) In the mini-retrosigmoid technique, image guidance is utilized to make a slightly S-shaped incision that exposes the transverse-sigmoid junction, with a small posterior craniectomy to allow a flat trajectory to the petrous face before turning medially into the CSF cisterns. Modified from: Rosario Van Tulpe, https://commons.wikimedia.org/wiki/File:SkullSchaedelSeitlich1.png

In the mini-retrosigmoid approach, the patient is typically positioned supine, with the head rotated to the contralateral side. The ipsilateral shoulder is never elevated, and bed rotation is used to obtain additional working angles. The head is also flexed anteriorly about 10° to achieve optimal ergonomic conditions for the surgeon. Lateral flexion is variable, depending on the desired working angle. Using image guidance, the incision is S-shaped and crosses over the transverse-sigmoid junction. The craniectomy is about 2 cm and exposes the transverse-sigmoid junction, with a small amount of posterior craniectomy needed to allow a flat trajectory to get to the petrous face and turn medially into the CSF cisterns. This is compared to the standard approach in Figure 4. The dural opening is then performed in a T-shaped fashion, and the operating microscope is subsequently brought into the field. It is critical to keep the dural opening small as this prevents cerebellar extrusion which may complicate a bigger opening. The cerebellum is retracted medially, and arachnoidal dissection is carried out to expose the cisterna magna and allow for CSF relaxation. Regardless of the tumor size, the surgeon will have unlimited time to get to CSF and drain it without the brain coming out, if the dural opening is small. Tumor dural attachments are coagulated, and the resection is carried out in the tentorium-V, V-VII, and VII-lower cranial nerve intervals. Cranial nerves are meticulously separated from the tumor, and the brainstem is dissected away from the lesion. Tumor extending into the cavernous sinus is left for adjuvant stereotactic radiosurgery. Usually the bone defect can be repaired with a burr hole cover.

### Tailored supratentorial

**Figure 5:**
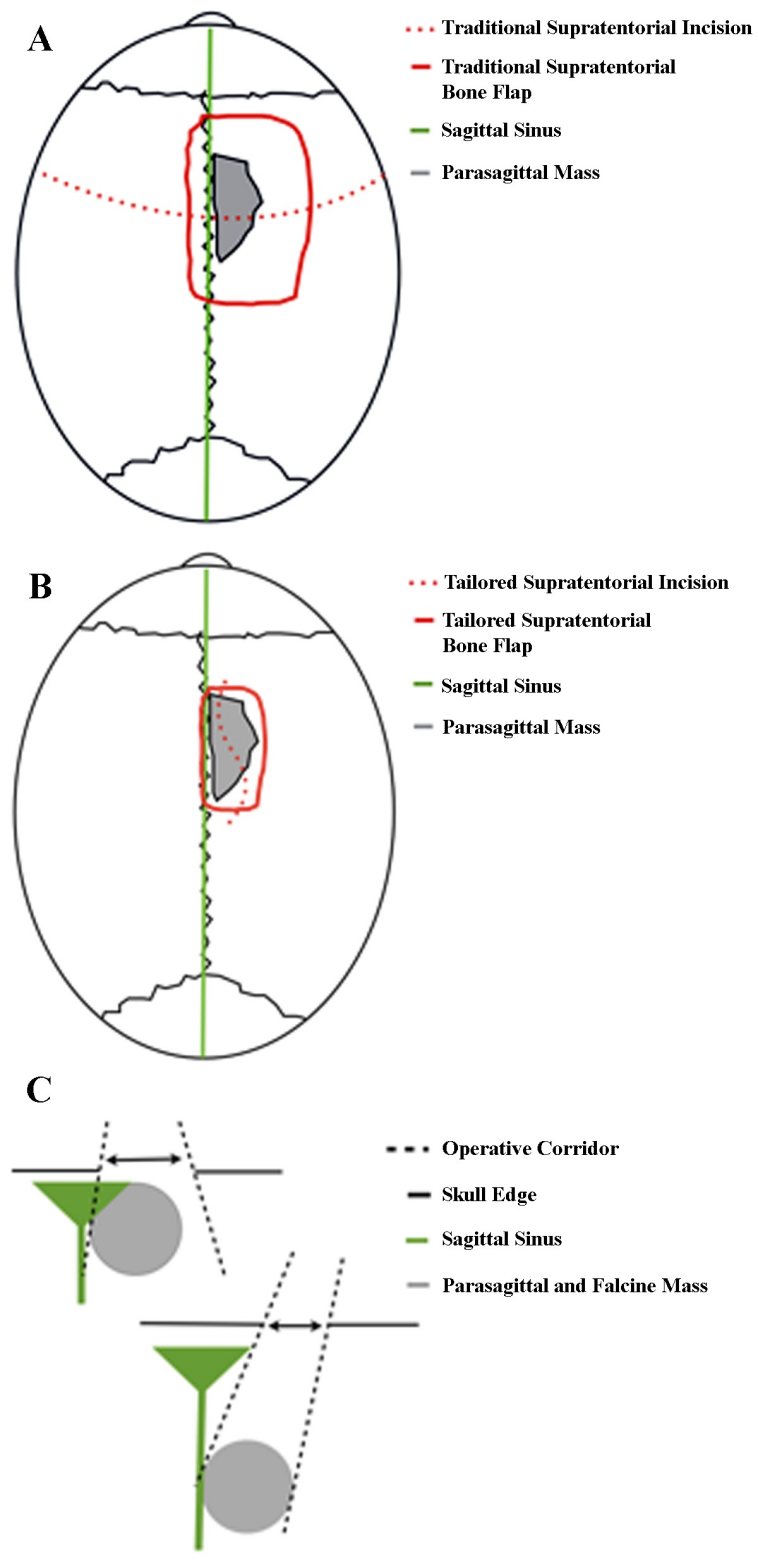
Standard supratentorial approach (A) vs. tailored supratentorial approach (B) The lesion(s) are measured in all dimensions with image guidance to plan a tailored supratentorial approach for meningiomas of the convexities, falx, or parasagittal regions. For this approach, the lesion(s) must be deep enough to be accessed through the keyhole. The keyhole concept is illustrated in (C).

Using image guidance, the lesion(s) are measured in all dimensions, and a miniature craniotomy is planned if the lesion is deep-seated enough to be visualized adequately with such an approach. Differences from a standard supratentorial approach are shown in Figure 5. Lesions located on the convexities, falx, and parasagittal regions were all resected in this fashion. Closure typically requires a dural graft and is otherwise straightforward.

## Results

Twenty-seven patients harboring 31 tumors were identified. Patient data are summarized in Table 1. The mean age of the cohort was 55 years (range, 31-72 years). Nineteen (70%) of the patients were female. The most common presenting complaint was visual loss occurring in 9 patients; vision loss resolved in 2 patients, improved in 5 patients, remained stable in 1 patient, and worsened in 1 patient. Six patients had cranial nerve palsies upon presentation; all but two of these deficits had improved or resolved at last follow-up. The median length of stay was 4 days (range, 1-20 days). Twenty-nine tumors were WHO Grade I on histologic examination, and two were WHO Grade II. Twelve patients received adjuvant radiation (44%). Simpson resection grade was as follows: Grade I = 8 patients; Grade II = 8 patients; Grade III = 1 patient; Grade IV = 14 patients; Grade V = 0 patients. Four patients suffered major complications. Four patients developed new cranial nerve deficits postoperatively, which were improving or resolved at last follow-up. One patient had tumor recurrence after 1 year and received another resection; there has been no other evidence of additional recurrence after 14 months.

**Table 1:** Case Summaries * Preoperatively-planned subtotal resection; Abbreviations: CN, cranial nerve; GK, gamma knife radiosurgery; IMRT, intensity-modulated radiation therapy; LINAC, linear particle accelerator-based radiation therapy; LOS, length of stay; VPS, ventriculoperitoneal shunt.

Patient Summaries											
	Age / Gender		Tumor Location	WHO Grade	Operative Time	Simpson Resection Grade	Preoperative Deficits (Postoperative outcome)	Major Complications	Minor Complications	LOS (Days)	Adjuvant Therapy
Supraorbital	53 / M		R planum sphenoidale/anterior skull base	I	4:38	I	None	Pneumocephalus, DVT/PE	-	3	-
	57 / F		L planum sphenoidale	I	4:09	II	L CN VIII (resolved)	-	-	2	-
	66 / M		Midline tuberculum sellae	I	12:47	II	Visual loss (resolved)	-	-	3	-
	60 / F		R posterior clinoid	I	9:49	IV	Visual loss (resolved)	-	-	6	IMRT
	58 / F		L medial third of sphenoid wing/optic canal	I	16:14	IV	Visual loss (improved)	-	-	5	IMRT
	41 / M		L olfactory groove/planum sphenoidale	II	15:50	IV	Visual loss (improved)	-	-	4	Proton
	53 / F		R anterior clinoid	I	3:03	II	Anisocoria, diplopia (resolved)	-	-	2	-
	54 / F		Midline olfactory groove/planum sphenoidale	I	2:55	II	None	-	-	3	-
	60 / M		L supraorbital	I	4:34	I	None	-	-	2	-
	63 / F		Midline tuberculum sellae	I	7:07	III	Visual loss (improved)	-	Small pseudomeningo-cele	4	-
Mini-Pterional	41 / F		L sphenoid wing (recurrent)	I	4:43	IV*	L complete visual loss (persistent)	-	-	4	GK
	72 / F		L sphenoid wing/cavernous sinus/complex skull base	I	13:52	I	L visual loss (improved)	-	-	15	-
Mini-Retrosigmoid	64 / F		R sphenopetroclival	I	9:46	IV*	Visual loss (worsened)	Perioperative R PICA infarct	-	20	LINAC
	46 / F		L posterior clinoid and petroclival	I	5:18	II	L CN IV palsy (resolved)	-	-	3	-
	62 / F		L cerebellopontine angle	I	6:10	IV	L CN VIII palsy (stable)	-	-	4	-
	51 / F		R petrous apex/complex skull base	I	5:52	I	Diplopia (improved), R CN V3 palsy (resolved)	-	CSF leak; Small pseudomeningo-cele	6	-
Tailored Supratentorial	46 / F		R frontal convexity	I	1:45	I	None	Infection	-	1	-
	63 / M		L falcine	I	6:42	II	None	-	-	2	-
	57 / F		L frontal convexity	I	2:41	I	None	-	-	4	-
	31 / F		L frontal convexity	I	3:02	IV	None	-	-	4	-
	61 / F		R falcine	I	7:13	IV	None	-	-	7	GK
	65 / M		R parasagittal	I	4:27	I	None	-	-	3	-
Combination	37 / F		L frontal convexity (tailored supratentorial)	I	11:17	II	None	-	CSF leak	2	-
			R frontal convexity (tailored supratentorial)	I		I					-
			Foramen magnum (retrosigmoid)	I	14:00	IV*		-	Decreased temperature sensation	8	GK
	62 / F		R falcotentorial (tailored supratentorial)	I	8:56	II	None	-	-	8	GK
			L transverse sinus/cerebellar (retrosigmoid)	II		IV					GK
			R jugular foramen (retrosigmoid)	I		IV*					-
	61 / M		Bilateral L > R sphenocavernous/petroclival (pterional + endonasal + retrosigmoid)	I	15:02	IV*	L visual loss (improved)	Hydrocephalus	-	12	GK
	57 / F		R sphenopetroclival (retrosigmoid + pterional)	I	9:50	IV*	R CN VI palsy (persistent)	-	-	4	GK
	53 / M		L sphenocavernous and infratentorial (pterional + endonasal)	I	12:48	IV*	None	-	V2 neuralgia	5	GK

### Approach selection and operative goals

The approach was chosen based on senior author preference and lesion size, location, invasion, and intimacy with surrounding critical structures. Although in most patients the goal of surgery was complete resection, subtotal resection was planned for 7 of the Grade IV patients (50%) due to clear involvement of the cavernous sinus in 5 patients, 360° encasement of the vertebral artery in 1 patient, and jugular foramen invasion in 1 patient. In remaining 7 patients, the preoperative goal was to attempt gross-total resection if safely possible. In these cases, intraoperative assessment of feasibility determined that the risk of postoperative deficits outweighed the advantage of pursuing aggressive resection.

**Figure 6:**
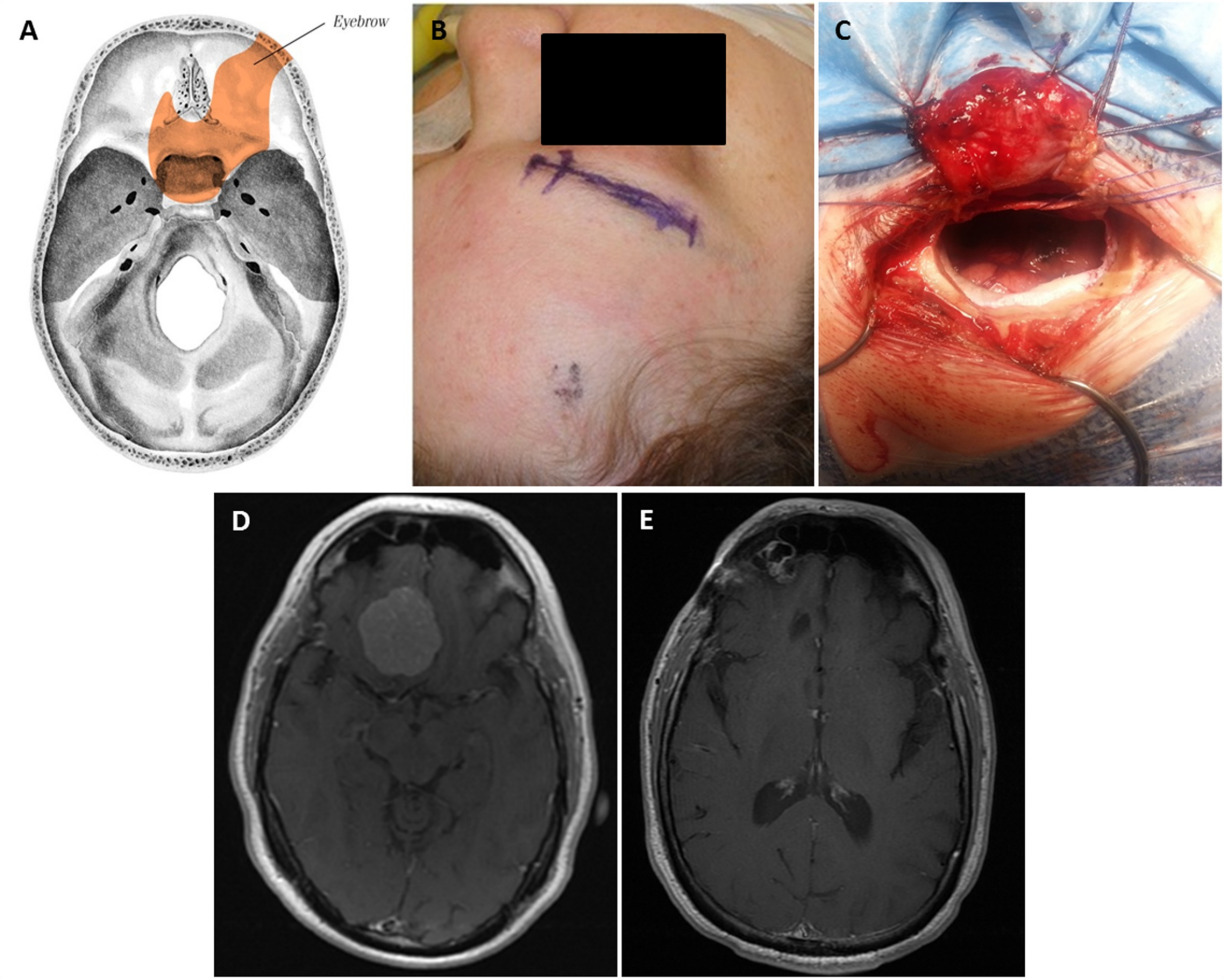
Supraorbital “Eyebrow” Keyhole Approach The skull base regions accessible with this approach (A). Planned incision (B) and craniotomy (C). Preoperative post-contrast axial T1 MRI shows right planum sphenoidale/anterior skull base meningioma (D). Postoperative axial MRI (E).

In this series, the supraorbital approach was utilized to resect meningiomas of the olfactory groove, planum sphenoidale, tuberculum sella, some tumors of the medial sphenoid wing, and posterior clinoid (Figure 6). In the 10 patients treated, 1 experienced pneumocephalus and 1 developed a small pseudomeningocele.

**Figure 7:**
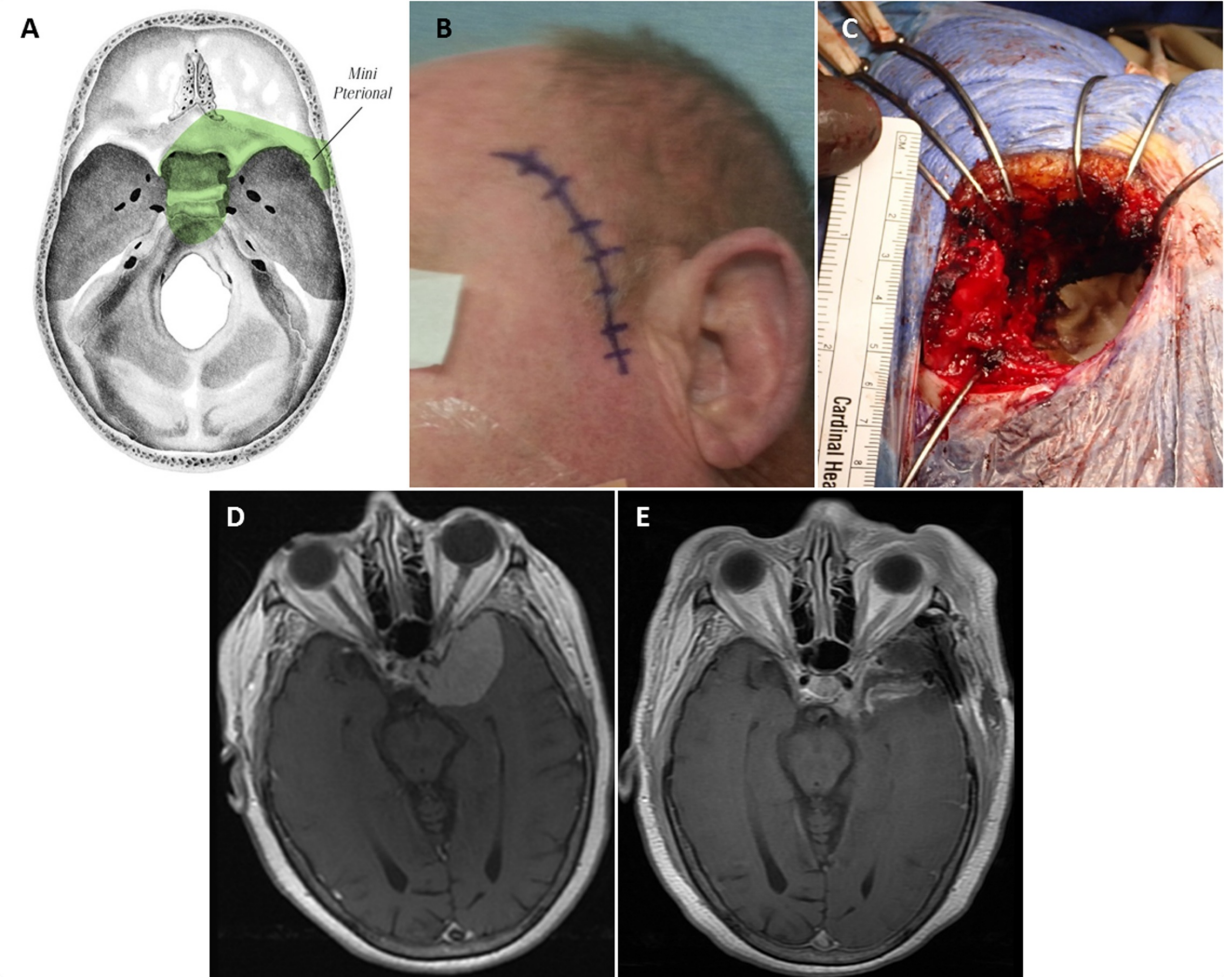
Miniature Pterional Keyhole Approach The skull base regions accessible through this approach (A). Planned incision (B) and craniotomy (C), which is approximately 5.5 cm in largest diameter. Preoperative post-contrast axial T1 MRI shows complex left sphenoid wing and complex skull base meningioma with cavernous sinus invasion (D). Postoperative axial MRI (E).

The mini-pterional approach was utilized in 5 cases involving the sphenoid bone (Figure 7). Two patients with sphenoid wing meningiomas were treated exclusively with this approach, and 3 patients with more complex lesions involving the sphenoid bone were treated with a mini-pterional craniotomy as part of a combination treatment. All 5 patients displayed cavernous sinus invasion on preoperative contrast-enhanced MRI.

**Figure 8:**
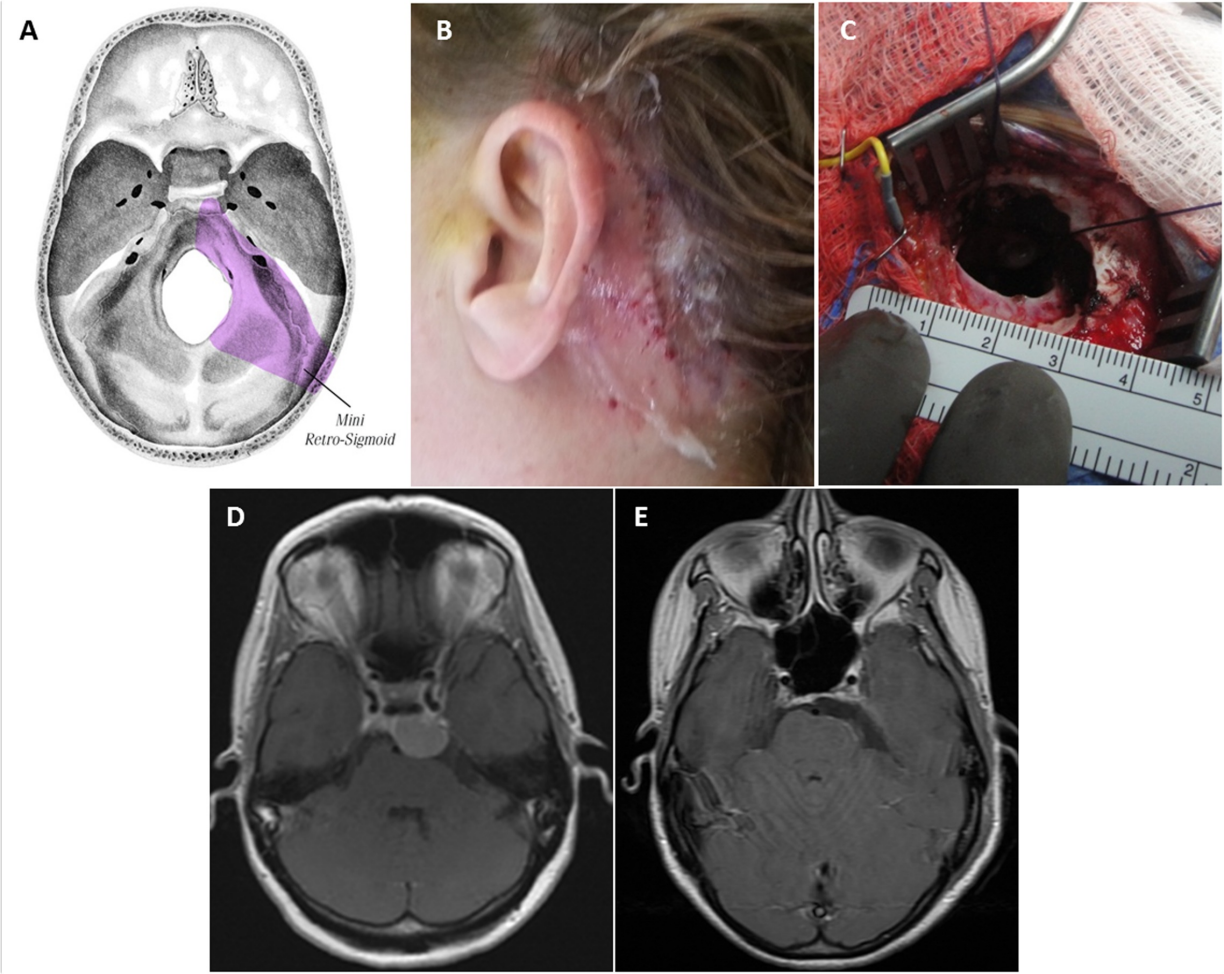
Miniature Retrosigmoid Keyhole Approach The skull base regions accessible through this approach (A). Postoperative incision site (B). Keyhole craniotomy less than 3 cm (C). Preoperative post-contrast axial T1 MRI shows complex left posterior clinoid/petroclival meningioma (D). Postoperative axial MRI (E).

The majority of posterior fossa meningiomas encountered during the study period were resected through the retrosigmoid approach, used to approach a total of 9 tumors. We have found that nearly every ventral posterior fossa lesion can be removed through a small opening if placed well. It is simple, fast, and very effective. This approach gives excellent visualization of the cranial nerves as they exit the brainstem and course to their various foramina, and was utilized for patients with sphenopetroclival, posterior clinoid/petroclival, petrous apex, and cerebellopontine angle meningiomas (Figure 8).

**Figure 9:**
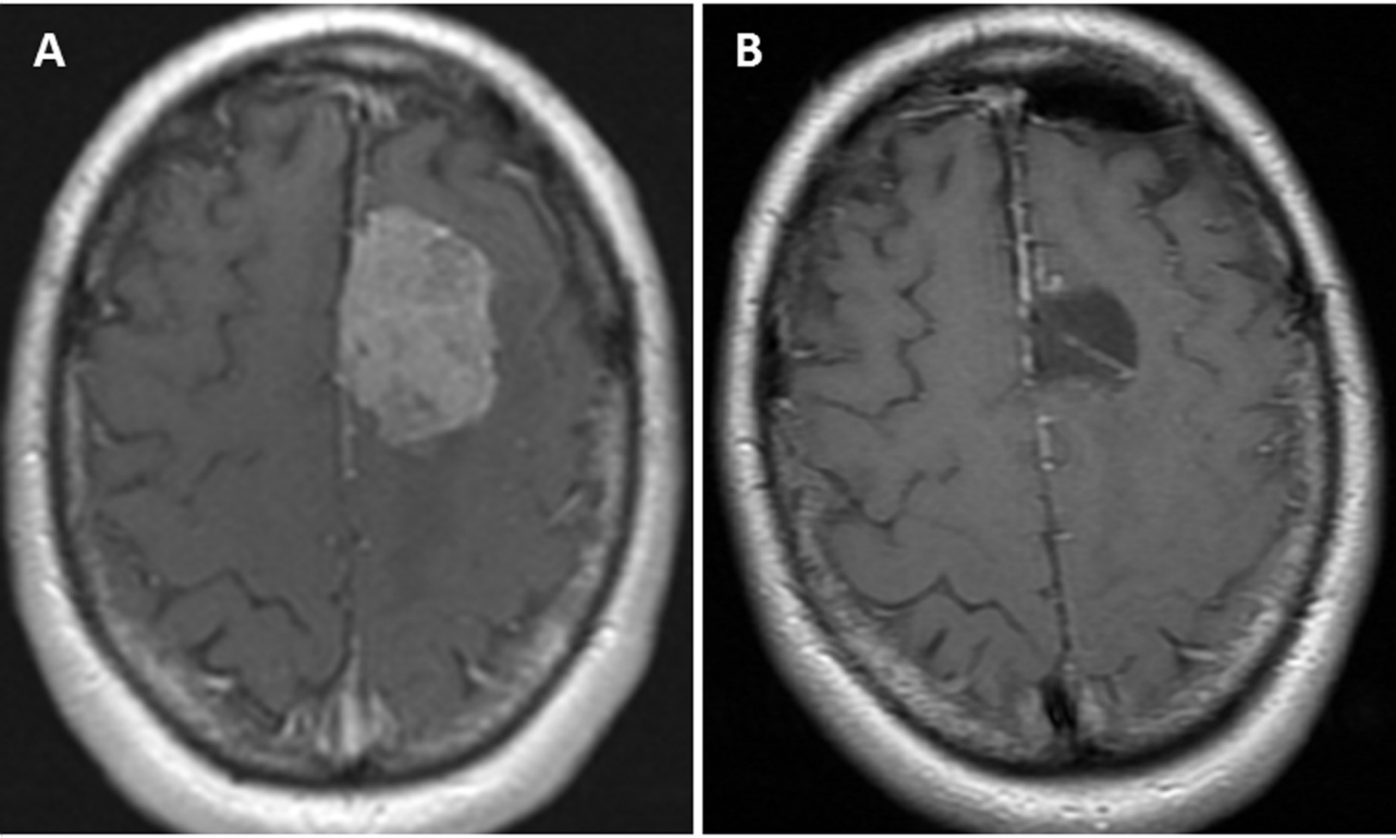
Tailored Keyhole Approach Preoperative post-contrast axial T1 MRI shows left falcine meningioma (A). Postoperative axial MRI shows complete resection (B).

We performed tailored supratentorial keyhole approaches for 9 patients with convexity and falcine meningiomas as an exclusive procedure (Figure 9) and for 2 patients as part of a combination of approaches to resect multiple lesions in each.

Five patients in our series were treated with a combination of approaches, either for multiple tumors (2 patients) or for complex skull-base lesions that that could not be adequately resected with a single approach (3 patients). These were performed either in the same operation or as a staged procedure. In 2 of the 3 multiple-approach cases, the endoscopic endonasal approach was used in addition to keyhole approaches. 

## Discussion

The supraorbital [3-7], mini-pterional [8-9], retrosigmoid [3, 10-12], and tailored supratentorial [2, 9-10] approaches are well-described, though to a lesser degree for resecting meningiomas. Additionally, there are limited data regarding the use of a comprehensive keyhole-based system in managing these lesions. The goal of keyhole neurosurgery is to achieve maximal efficiency with minimal trauma to the patient, mainly by limiting brain exploration and retraction [1]. With proper patient positioning and preoperative planning, utilization of gravity for brain self-retraction, and early CSF drainage to promote brain relaxation, most lesions can be accessed with miniature craniotomies.

The supraorbital approach allows a corridor to much of the anterior and middle cranial base and minimizes or eliminates frontal lobe retraction while exposing lesions of the sella, sphenoid wing, posterior clinoid, and other regions of the skull base. Neurovascular structures are also well visualized, and the cosmetic result is optimal. The mini-pterional approach provides early visualization of the optic nerves and anterior circulation vessels while minimizing exposure and risk to unneeded parts of the frontal lobe. The advantages of a conventional pterional approach are preserved, while unnecessary exposure of frontal and temporal lobes is avoided. A retrosigmoid keyhole approach provides a route to the cerebellopontine angle, upper and middle clivus, and with minor variation may provide an avenue to the lower clivus and foramen magnum. In cases with large tumors, the lesion’s mass effect creates a working space between the brainstem, cranial nerves, and the skull base [13]. After early and sufficient CSF drainage and opening of the subarachnoid cisterns, use of brain retractors is always unnecessary.

Although Simpson Grade IV resections occurred in 14 patients, half were planned and the rest were due to tumor encasement of vital neurovascular structures. In the authors’ eyes, gross-total resection in these patients would have been prohibitively difficult regardless of the size of the approach, and the resultant risk of morbidity or mortality was not deemed worthwhile when weighed against adjuvant radiation for residual tumor. Several authors have found judicious resection of complex skull base meningiomas (with or without stereotactic radiosurgery) results in acceptable long-term functional outcomes and is a viable alternative to pursuing gross-total resection [11, 14-15].

## Conclusions

Patients with intracranial meningiomas in a variety of locations can be treated successfully using keyhole techniques. The patient cohort experienced satisfactory results in the perioperative period and through the time of last follow-up. 
